# Sulvanites: The Promise at the Nanoscale

**DOI:** 10.3390/nano11030823

**Published:** 2021-03-23

**Authors:** Roberto Prado-Rivera, Chen-Yu Chang, Mimi Liu, Cheng-Yu Lai, Daniela R. Radu

**Affiliations:** Department of Mechanical and Materials Engineering, Florida International University, Miami, FL 33199, USA; rprad009@fiu.edu (R.P.-R.); chechang@fiu.edu (C.-Y.C.); mliu@fiu.edu (M.L.); clai@fiu.edu (C.-Y.L.)

**Keywords:** sulvanite, solid-state synthesis, solution-phase synthesis, electronic structure, optical bandgap, elastic properties, thermodynamic properties

## Abstract

The class of ternary copper chalcogenides Cu_3_MX_4_ (M = V, Nb, Ta; X = S, Se, Te), also known as the sulvanite family, has attracted attention in the past decade as featuring promising materials for optoelectronic devices, including solar photovoltaics. Experimental and theoretical studies of these semiconductors have provided much insight into their properties, both in bulk and at the nanoscale. The recent realization of sulvanites at the nanoscale opens new avenues for the compounds toward printable electronics. This review is aimed at the consideration of synthesis methods, relevant properties and the recent developments of the most important sulvanites.

## 1. Introduction

Ternary copper chalcogenides have recently attracted attention due to their interesting electronic and optical properties. In particular, the class of compounds Cu_3_MX_4_ (M = V, Nb, Ta; X = S, Se, Te), known as sulvanites or sulvanite-type compounds, are *p*-type intermediate band gap (IB) semiconductors that have shown promise in optoelectronics due to their large ionic mobility [1,2], high absorption in the visible and UV range [3,4], low hole effective mass [5], and optical band gaps suitable for their use as an absorber layer in thin film solar photovoltaics [6]. Such characteristics have driven several studies into the fabrication of the sulvanite-type compounds as thin-film photovoltaics (PV) [4,7,8,9,10] and photocatalysts for H_2_ production [11]. The fabrication of sulvanite nanocrystals with tunable size and properties [9,12] became an important endeavor, toward enabling the fabrication of devices on flexible substrates.

The sulvanite-type compounds also show appeal in their elemental composition. These compounds are largely comprised of Earth-abundant, sustainable, and non-toxic elements. This is particularly advantageous when seeking alternatives to PV materials that are known to have expensive production costs and include toxic and scarce elements, notably CdTe and Cu(In_x_Ga_1−x_)S_2_ (CIGS). Furthermore, the synthesis of the sulvanite-type compounds proved facile both via solid-state and solution-phase methodologies.

This review discusses several key characteristics of the sulvanites, first introducing the crystal structure, followed by the preparation of these compounds in their various forms exhibited in the literature. Afterwards, a summary of key properties from both experimental and theoretical studies will be provided, including the electronic structure and optical, elastic, and thermodynamic properties. Lastly, recent developments in the sulvanite-type compounds will be presented.

## 2. Sulvanites Structure

**Figure 1:**
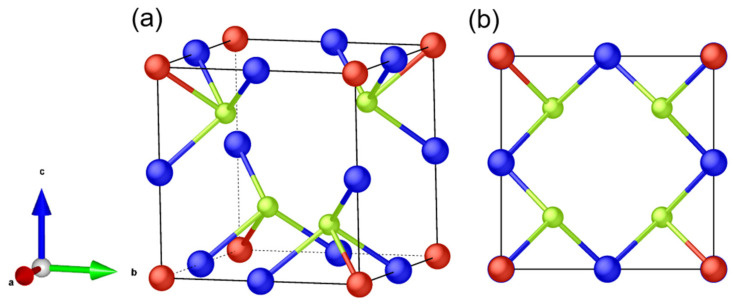
(**a**) The unit cell of Cu_3_MX_4_. (**b**) The unit cell of Cu_3_MX_4_, as seen from <100> direction. (Red is attributed to *M* transition metal ion, blue represents Cu ions, light green is attributed to chalcogen ions.)

The first complete description of the crystal structure of sulvanite was done by Pauling and Hultgren, who performed oscillation and Laue photographs on a sample of natural crystal Cu_3_VS_4_ [13]. It was found that Cu_3_VS_4_ crystallizes in a simple cubic structure with space group $T_{d}^{1}-P\bar{4}3m$ (No. 215). Other studies confirmed this cubic structure for the rest of the Cu_3_MX_4_ compounds [14,15,16,17,18,19,20,21]. As shown in Figure 1, the corners of the unit cell are occupied by an *M* transition metal ion at Wykoff position 1*a*, a Cu ion lies at the center of the edges at Wykoff position 3*d*, and the chalcogen ions are placed at Wykoff position 4*e*. The structure entails tetrahedral coordination between the metal ions and four neighboring chalcogen ions. The tetrahedra are organized in such a way as to allow for empty channels through the <100> directions of the lattice. These empty channels have been shown to facilitate high mobility of interstitial ions [1,2,22], and theoretical predictions point to a high power factor in thermoelectric applications [23]

The cell parameters range from 5.370 Å (Cu_3_VS_4_) to 5.930 Å (Cu_3_TaTe_4_). As the *M* ion changes from V, to Nb and Ta, there is an increase in the cell parameter, consistent with the increase in the size of the *M* ion. A similar trend is found when the chalcogen ions are substituted, with S producing the smallest cell parameter, Se—the intermediate and Te—the largest. Remarkably, despite the range of cell parameters and choices in the *M* and *X* ions, the sulvanite-type compounds offer the possibility for heteroepitaxial thin film growth on silicon due to good lattice constant matching, which is advantageous for the fabrication of electronic devices [7].

A summary of the cell parameters of each compound is presented in Table 1 (references are assigned to each compound in the table) which includes both experimental results and theoretical predictions of the cell parameters from density functional theory (DFT). The following abbreviations are used in Table 1 and Table 2: PBE, PBEsol, and HSE06 are functionals used within DFT; PBE and PBEsol stand for the Perdew–Burke–Ernzerhof functionals and are known as generalized gradient approximation type (GGA) that approximate the total energy as a sum of the electron density and its gradient. HSE06 is an abbreviation for the Heyd–Scuseria–Ernzerhof (2006) functional. HSE06 belongs to a class of functionals known as “hybrid” functionals that combine GGA and another approximation, in this case a parameterized Hartree–Fock energy approximation.

## 3. Preparation of Cu_3_MX_4_

Cu_3_VS_4_ is found in nature as a mineral, called sulvanite, and the mineral name remained the generic name for the Cu_3_MX_4_ (M = V, Nb, Ta; X = S, Se, Te) class. No other sulvanite is found in nature, and methods have been developed to produce them. Cu_3_MX_4_ can be made primarily by two approaches: solid-state or solution-phase synthesis. Of the two, the solid-state synthesis method was first reported in 1967 [39]. Despite the long reaction times and high reaction temperature, this method is versatile, and each compound can be made from its corresponding elements via the solid state reaction approach. On the other hand, the solution-phase synthesis is a more energy-efficient approach, and, due to the ability to control reaction kinetics [40], it also permits control over the size and shape of the synthesized materials, which are obtained in nanoparticle form. However, the method is rather sensitive to reaction conditions, due to reactivity of nanoscale particles, and the variation in synthesis approaches was reported from one sulvanite to another. A summary and discussion of existing synthesis methods currently found in the literature is presented as follows. 

### 3.1. Solid-State Synthesis

Two solid-state techniques are commonly employed and entail the following: (i) mixing of pure elements (Cu, M, and X) in a stoichiometric ratio [4,18,20,21,34,35,38,39], or (ii) mixing two binary compounds [25,30,41] (the corresponding copper chalcogenide and metal chalcogenide) in a stoichiometric ratio. The mixing is followed by an annealing process at around 800 K, and it requires from several days to a few weeks. The solid-state route is versatile as it can be used to synthesize almost all the compounds in the Cu_3_MX_4_ family. As it is provided, a solid solution could be formed by mixing different transitional metals in group 5 [11] and/or chalcogenides [23,34], and the successful attempts were made to create mixed compositions. This approach enables the manipulation of the cell parameters and leads to a band gap change in the resulting compound, offering flexibility in the control of the desired optoelectronic properties.

### 3.2. Pulsed Laser Deposition

A two-step pulsed laser deposition method can be used to produce Cu_3_TaX_4_ thin films, as demonstrated by Tate and coworkers [42]. This method is first using a laser to deposit a binary metal multilayer stack onto the substrate, layer-by-layer. This step is followed by a heating process and sulfurization to allow for the diffusion of the metal atoms and to introduce the sulfur atoms that give rise to the ternary sulvanite. The same group reported thin-film Cu_3_TaS_4−x_Se_x_ and Cu_3_TaSe_4−x_Te_x_ solid solutions prepared using a similar deposition method, by annealing together the as-prepared Cu_3_TaSe_4_ thin film with Cu_3_TaS_4_ or Cu_3_TaTe_4_, respectively, at around 600 °C for 2 h [7].

In the work done by Lv et al. [4], powder Cu_3_VS_4_ was made and pressed into a pellet. This Cu_3_VS_4_ pellet was then used as the pulsed laser deposition target to fabricate a Cu_3_VS_4_ nanofilm with 220 nm thickness. 

### 3.3. Solution-Phase Synthesis

The solution-phase synthesis methods allow for the control of particle size and morphology, which conventional solid-state synthesis fails to achieve. Thus, the past two decades have seen a significant number of literature reports on nanomaterials synthesized through the solution-phase methods. A common feature of many of these syntheses is the hot injection method, which involves the use of long alkyl chain, high-boiling point solvents. Among these solvents, oleylamine (C_18_H_35_NH_2_, (Z)-Octadec-9-enylamine, OLA) is one of the most common, and it acts as a solvent, growth control agent and caping ligand on the surface of the formed nanoparticles [43]. The presence of OLA largely decreases the required formation energy for the sulvanite material by stabilizing the intermediates in a complex form. As a result, the solution-phase method can be conducted under a much lower reaction temperature (270 °C) comparing to the solid-state synthesis. The first report on the synthesis of Cu_3_MX_4_ nanoparticles through a solution-phase process was for spherical Cu_3_VS_4_ nanoparticles [44]. However, the synthesized product did not have good crystallinity, and a post-annealing process was utilized to improve the crystallinity of the Cu_3_VS_4_ nanoparticles. Later, the syntheses of cubic Cu_3_VS_4_ nanocubes [12], Cu_3_VSe_4_ nanocubes [9], and Cu_3_VSe_4_ nanosheets [8] were reported. Extending the reaction time leads to the facile preparation of high purity Cu_3_VS_4_ and Cu_3_VSe_4_ without the post-annealing treatment. Mantella et al. [12] reported that the size of the synthesized Cu_3_VS_4_ nanocrystals could be controlled through the reaction temperature. At 250, 260, and 280 °C, the nanocrystals were produced with an average size of (9 ± 1.3), (12 ± 1.4), and (17 ± 2.2) nm, respectively. 

The shape control of synthesized nanoparticles requires the fine tuning of particle surface parameters, which is accomplish by using combinations of surfactants. For example, well-defined, cubic-shaped Cu_3_VS_4_ were obtained by using a mixture of OLA, octadecene, and trioctylephosphine. Another example is the use of trioctylephosphine oxide, oleic acid, and OLA as surfactants for the synthesis of Cu_3_VSe_4_ nanocubes. Both of these examples follow similar procedures: two cation precursors (Cu and V) are first mixed, then chalcogenide precursors (S or Se) are injected to start crystal growth. 

To elucidate the mechanism of nanocrystal formation, Mantella et al. conducted a time dependence study which consisted of removal and analysis of aliquots from the reaction mixture by XRD and TEM [12]. The XRD analyses revealed that covellite CuS and chalcocite Cu_2_S are formed first and are the major crystalline compounds at the reaction onset. Cu_x_S peaks start to decrease as the Cu_3_VS_4_ characteristic peaks start to appear after five minutes in the reaction progression. The TEM images show a clear metamorphosis of the hexagonal platelet seeds into the cubic Cu_3_VS_4_, confirmed by the elemental mapping. A control experiment consisting of the reaction of pre-synthesized Cu_2−x_S with a vanadium precursor, led to the successful formation of the ternary Cu_3_VS_4__,_ proving the proposed mechanism. However, unlike the soluble precursors (CuCl for Cu and 1-dodecanethiol for S), the Cu_2−x_S precipitate and aggregate easily, leading to a wide particle size distribution in the final product. 

Early work reported by Swihart et al. presented the preparation of ternary chalcogenides CuInS_2_, CuGaS_2_, CuFeS_2_, Cu_2_SnS_3_, and Cu_2_GeS_3_ in nanoplatelets morphology through incorporating the third cation (In^3+^, Ga^3+^, Fe^3+^, Sn^4+^, and Ge^4+^, respectively) into the formed covellite CuS templates during colloidal synthesis [45]. Although the methodology of incorporating cations into the CuX template can be applied across a wide variety of crystal structures to make well-defined nanoparticles and nanoplatelets [46,47,48], this pathway leads to nanoparticles aggregation in the case of sulvanites [12].

However, when using vanadium sulfide as a precursor, a very interesting result was observed [8]. Herein, Liu et al. used a cascade synthesis strategy where vanadium and selenium precursors were allowed to react first, forming VSe_2_ nanosheets, as VSe_2_ is a 2D transition metal dichalcogenide (TMD). The copper precursor was then injected into the VSe_2_ suspension leading to the formation of Cu_3_VSe_4_. Remarkably, this approach directs the ternary Cu_3_VSe_4_ nanocrystal to form at the expense of the 2D VSe_2_ nanosheet template through a cation exchange process. The resulting product was able to conform to the morphology of the 2D template, resulting in a quasi-2D morphology, an interesting result for a purely cubic material in bulk. Both synthesis routes, using either CuX or MX_2_ as template, can lead to the formation of ternary Cu_3_MX_4_ compound. However, due to the templating effect [49], the resulting product can vary in its morphology.

Furthermore, colloidal synthesis enables the preparation of hybrid shapes, such as core-shell structured nanoparticles. In Y. Liu’s work [24], the Cu_3_VS_4_/CdS core-shell nanocrystals were prepared by coating a layer of CdS onto cubic-like Cu_3_VS_4_ nanocrystals. Although the colloidal method has much more flexibility with size and morphology control, there are few reports of the preparation of telluride Cu_3_MX_4_ compounds through colloidal solution-phase synthesis. As mentioned above, elemental chalcogenides are commonly employed as the chalcogen source due to their readily preparation by dissolution in a variety of nonpolar, aliphatic solvents. When elemental chalcogenides are used in colloidal synthesis, they are typically dissolved in common solvents, such as amine, alkenes, thiols, phosphines, to form soluble chalcogenide precursors [50]. However, the elemental tellurium exhibits an extremely low solubility in most aliphatic solvents, which is likely due to its lower reduction potential [50,51,52]. Phosphine ligands, which could be used as solvent for elemental tellurium, are air sensitive and expensive, limiting their extensive application in the colloidal synthesis of Cu_3_MTe_4_ compounds. Furthermore, the reactivity of elements decreases from lightest to heaviest in the same group; tellurium, with a larger atomic number could react with other elements but not as readily as sulfur and selenium do. Other tellurides have been prepared through solvothermal synthesis and resulted in nanosheet morphologies, including the quaternary AgPb_10_BiTe_12_ [48]; however, NbCl_5_ and TaCl_5_ which are commonly used as starting materials in sulvanite synthesis readily hydrolyze in air, potentially hindering the preparation of Cu_3_MTe_4_ compounds by the solvothermal method.

Despite surfactants being widely used in solution-phase synthesis, understanding their role in the reaction is still challenging since most reactions involve more than one surfactant, making it hard to decouple their contributions. Additionally, the same surfactant may act differently in other reactions, given the chemical nature of the precursors used. For instance, 1-dodecanethiol, when use in the synthesis of Cu_3_VS_4_ nanocubes, acts as the sulfur source, while in the synthesis of Cu_3_VSe_4_ nanosheet, it only acts as a surfactant to stabilize the selenium surface defects. 

The first synthesis of the tantalum sulvanites was demonstrated by Liu et al. via the aforementioned cascade synthesis strategy. The successfully preparation of the tantalum sulvanites Cu_3_TaS_4_ and Cu_3_TaSe_4_ nanocrystals with prismatic morphology was reported in 2021 [10]. By tailoring reaction conditions, Cu_3_TaSe_4_ core-shells with spherical nanostructures were also synthesized by the same group through the hot-injection method [10].

In summary, the solution-phase method provides excellent control over the size and shape of sulvanite nanocrystals and the syntheses reported to date resulted in various size, shape, morphology, and crystallinity of the Cu_3_MX_4_ nanoparticles. In the solution-phase approach, surfactant selection affected the growth of the nanocrystals with potential for future studies regarding Cu_3_MX_4_ nanoparticles with narrow size distribution. 

## 4. Electronic Structure and Properties of Sulvanites

**Figure 2:**
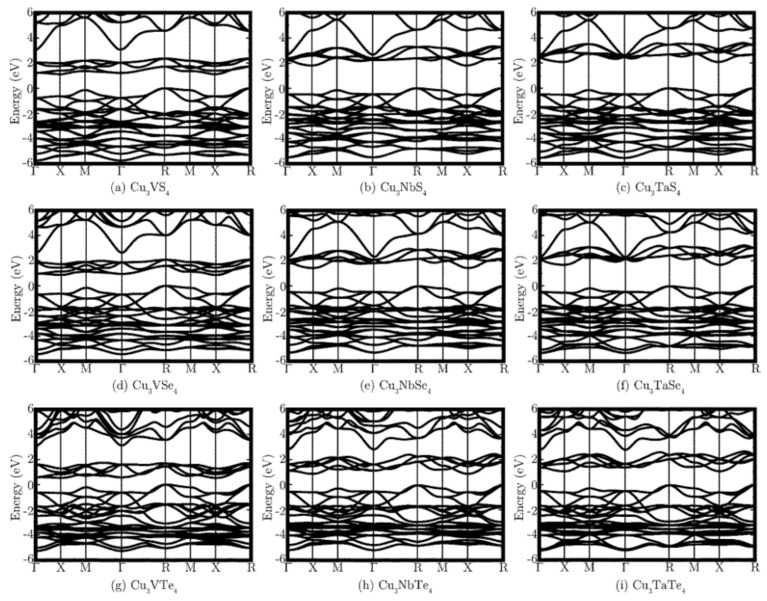
Electronic band structure of the sulvanite family of compounds calculated using PBEsol + U. The valence band maximum is taken to be zero. Reprinted with permission from [5], Copyright © 2015 Royal Society of Chemistry (RSC).

There have been several studies investigating the electronic structure of the sulvanite compounds using first-principles calculations [3,5,6,27,29,31]. From these studies, it has been shown that the compounds are indirect band gap semiconductors, and a wide range of band gaps was identified for sulvanites. As shown in Figure 2, the fundamental indirect band gap has its valence band maximum (VBM) placed at the *R* (1/2, 1/2, 1/2) symmetry point and the conduction band minimum (CBM) is at the *X* (0, ½, 0) symmetry point. The direct transition location depends on the transition metal of the compound [5,6]; for the Nb and Ta systems, it is located at *X*, while that of the V system lies at *M* (1/2, ½, 0). The CB edge is composed mostly of the transition metal *d*-states, which leads to increases in the energy when heavier transition metal ions are considered. This is recognized by the increase in the band gap as one goes from V to Nb to Ta. The VB edges are a hybridization of Cu *d*-states and chalcogen *p*-states. These chalcogen *p*-states have the effect of decreasing the value of the band gap by elevating the valence band edge as the chalcogen becomes heavier. The shape of the band structures is very similar to each other across all functionals used, with the only notable difference found in the energy of the conduction band being shifted depending on the functional.

Experimental and DFT band gaps are given in Table 2 (references are assigned to every compound). Of the available measurements, Cu_3_VS_4_ has the smallest of the experimental band gaps at 1.3–1.55 eV [4,11,25], while Cu_3_TaS_4_ has the largest at 2.70–2.83 [7,11]. The other experimental values are 2.50–2.60, 2.14–2.20, and 2.35–2.43 eV for Cu_3_NbS_4_ [11,34], Cu_3_NbSe_4_ [34,53], and Cu_3_TaSe_4_ [7,10,53], respectively. From these measurements, the band gaps are seen to vary by enlarging the ionic radius of the chalcogen (transition metal) ion, as predicted by computational studies. Missing from these experimental measurements, however, are the Te-sulvanites. The DFT calculations reveal that the substitution of S or Se with Te produces a band gap that is the lowest in the chalcogen series, so it is likely that experimental values would follow the same trend. In many of the related studies, GGA functionals give indirect band gaps that are often under their experimental partners while more advanced functionals and methods such as hybrid functionals and quasiparticle considerations lead to overestimations. As experimental band gap measurements come primarily from optical spectroscopy, the PBEsol + U calculated optical band gaps were determined by Kehoe and coworkers to best replicate the available data [6]. There is a good agreement for PBEsol + U calculated optical band gaps of the Ta compounds, 2.60 eV for Cu_3_TaS_4,_ and 2.22 eV for Cu_3_TaSe_4_. While not as close, the cases involving M = V, Nb, and PBEsol + U do give decent results as well, namely 1.72 eV (Cu_3_VS_4_), 2.31 eV (Cu_3_NbS_4_), and 1.95 eV (Cu_3_NbSe_4_). Espinosa-Garcia and coworkers came to a similar conclusion for PBEsol + U in accurately determining the band gaps of these compounds, which seems to be counterintuitive when comparing to the more sophisticated GW approximation used in the study. They also noted that past studies have only produced off-stoichiometric sulvanite crystals, which is not properly represented in theoretical studies. It was postulated that intrinsic defects within the material affect the band gaps as measured by optical spectroscopy, something that can be accounted for if excitonic effects are considered.

The hole effective masses were determined to be greatly impacted by the choice in the chalcogen element of the compound, decreasing in value when going from S to Se to Te [5]. Cu_3_MTe_4_ was calculated to possess the lowest hole effective mass, ranging between 0.607 m_h_* to 0.648 m_h_*, indicating the likelihood for high hole mobility. The calculations implemented by Li et al. [57] show similar results for the sulfide and selenide sulvanites, with the average hole effective masses being 0.73 m_h_*, 0.76 m_h_*, 0.93 m_h_*, and 0.94 m_h_* and the average electron effective masses being 1.18 m_e_*, 1.30 m_e_*, 1.40 m_e_*, 1.43 m_e_* for Cu_3_NbSe_4_, Cu_3_TaSe_4_, Cu_3_NbS_4_, and Cu_3_TaS_4_, respectively. The low effective masses were noted by Li et al. to aid in photocatalytic reactions. 

The carrier mobilities, as calculated by Li et al. using an empirical formula, were shown to be high. The mobility of the electrons for Cu_3_NbS_4_ and Cu_3_NbSe_4_ was determined to be 27.49 cm^2^/(V·s) and 39.55 cm^2^/(V·s), respectively. For the compounds Cu_3_TaS_4_ and Cu_3_TaSe_4_, the electron mobility was 25.66 cm^2^/(V·s) and 30.41 cm^2^/(V·s), which are slightly smaller than the values of the Nb counterparts. It can also be seen that the mobility increases with the chalcogen element as S to Se. The mobility of the holes is much higher than the values found for the electron mobility; the hole mobilities were determined to be 101.13, 163.97, 96.91, and 145.95 cm^2^/(V·s) for Cu_3_NbS_4_, Cu_3_NbSe_4_, Cu_3_TaS_4_, and Cu_3_TaSe_4_, respectively. By comparison, the hole mobility of polycrystalline Cu_3_TaS_4_ films from Hall measurements was found by Tate et al. [42] to be between 0.2 and 0.4 cm^2^/(V·s), much lower than that calculated by Li et al. However, Tate et al. postulated that the low mobility is likely due to grain boundaries and is possibly contact-limited as measurements made on BaCuChF (Ch = S, Se, Te) and BiCuOSe films yielded mobilities ten times larger within the same experiment.

The measurements first made by Arribart et al. [1] found the hole mobility of Cu_3_VS_4_ to be about 4 cm^2^/(V·s) and the electronic conductivity at room temperature to be 10^−3^ to 10 S/cm. Thin films of Cu_3_VS_4_ by Lv et al. [4] show a conductivity of about 1.88 S/cm, well within the range as given by Arribart et al. The other sulvanite-type compounds also fall within or near this range as well. The temperature-dependent electrical conductivity of Cu_3_NbS_4_ and Cu_3_NbSe_4_ were measured to be between 3.4 S/cm (598 K) to 14 S/cm (306 K) and 130 S/cm (598 K) to 202 S/cm (323 K), respectively [34], decreasing in value with temperature. The Van der Pauw measurements at room temperature carried out by Hersh yield conductivities of 0.255 S/cm and 1.67 × 10^−3^ S/cm for Cu_3_NbS_4_ and Cu_3_NbSe_4_, and 0.149 S/cm and 3.13 × 10^−3^ S/cm for Cu_3_TaS_4_ and Cu_3_TaSe_4_ [55], which are on the lower end of the range as given by Arribart et al. The conductivity of Cu_3_TaS_4_ was measured by Hersh and it is lower than the 1 S/cm for thin-film Cu_3_TaS_4_ [42]. The electrical conductivity of the sulvanite-type compounds varies approximately between the order of 10^−3^ and 10^2^ S/cm, the upper end of which is promising for optoelectronic applications. The thermoelectric figure of merit, ZT of *p*-type Cu_3_TaTe_4_ can reach ∼3 at 1000 K given its low lattice thermal conductivity (κ_l_) (0.38 W m^−1^ K^−1^). Interestingly, the lattice thermal conductivity is reduced to 0.17 W m^−1^ K^−1^ through 1 GPa pressure, which, due to phonon softening and strengthening of the acoustic and the optical phonon interactions, leads to a remarkable ZT of 5.368 at 1000 K.

## 5. Optical Properties

**Figure 3:**
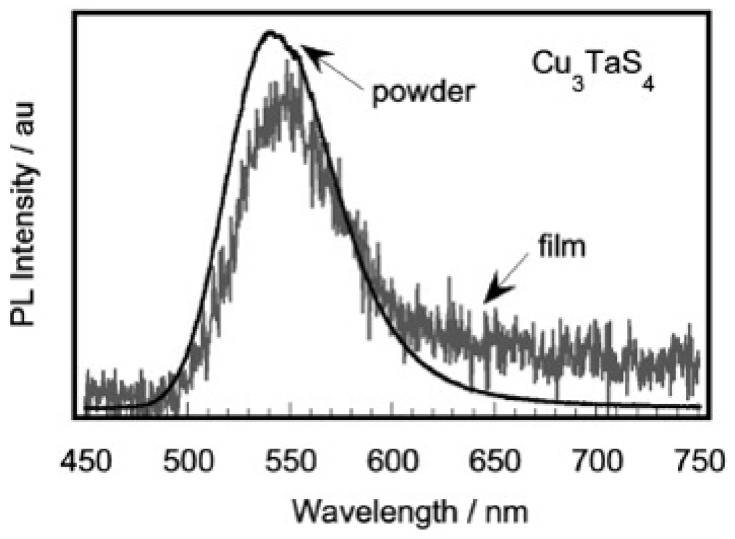
Photoluminescence spectra for Cu_3_TaS_4_ powder and thin film. Reprinted with permission from ref. [7], Copyright 2008 Elsevier B.V.

The large optical band gaps of these materials, particularly that of Cu_3_TaS_4_, has led to a few studies into their potential as transparent conductors. The measurements of Cu_3_TaS_4_ thin films [7,42] show the transmission *T* and reflection *R* coefficients to have average values of 53% and 22%, respectively, over the visible range, indicating that this material is mostly transmitting within this region. The transmission coefficient is seen to increase with wavelength up to approximately 80% as it approaches the infrared region. The refractive index was found from optical interference patterns to decrease from 2.45 to 2.19 within the 477–852 nm range and by prism coupler to be 2.30. The Cu_3_TaSe_4_ crystals prepared by Nitsche and Wild [39] exhibited similar yet larger values of the refractive index and transmission. Depending on the thickness, the refractive index of Cu_3_TaSe_4_ crystals ranged between 2.75 to 2.85, and the transmission measured up 65% within the infrared region. When illuminated by UV light, both Cu_3_TaS_4_ and Cu_3_TaSe_4_ exhibit photoluminescence, with maximum PL intensities positioned at 543 and 623 nm. Similar PL results were obtained for Cu_3_TaS_4_ in an early study, while also noting green photoemission for the <440 nm light source, as shown in Figure 3 [55]. In 2021, Liu et al. presented that the synthesized Cu_3_TaS_4_ nanocrystals possess a similar PL spectrum, which exhibited characteristic emission peaks at 486.3 and 531.4 nm when using 360 nm as the excitation wavelength [10]. This same study postulated that the source of the photoemission was due to Cu vacancies near the valence edge and, later, demonstrated that the photoemission intensity increased in Cu deficient samples.

**Figure 4:**
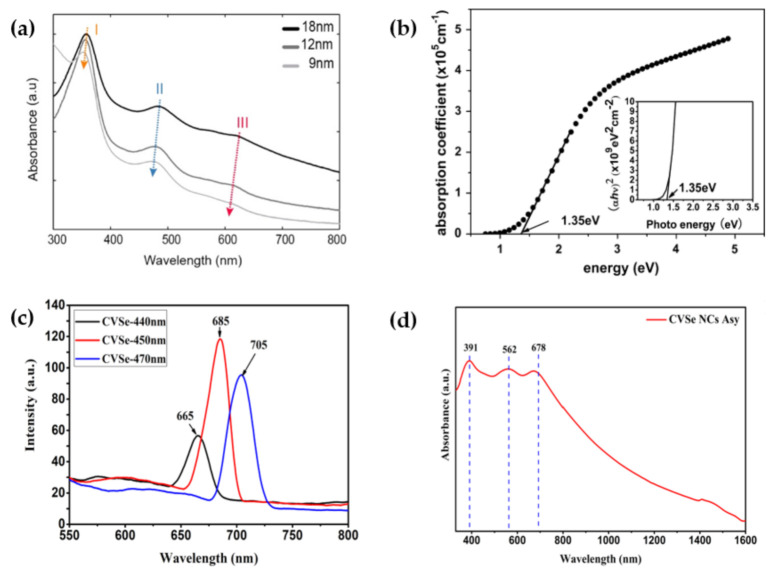
(**a**) UV-visible absorption spectra of Cu_3_VS_4_ nanocrystals of differing sizes. Reprinted with permission from ref. [12], Copyright 2019 American Chemical Society. (**b**) Absorption coefficient α versus the photon energy of Cu_3_VS_4_ nano-thin films. Reprinted with permission from ref. [4], Copyright 2012 Elsevier B.V. (**c**) Photoluminescence of Cu_3_VSe_4_ nanocrystals at different excitation wavelengths (440, 450, and 470 nm). Reprinted with permission from ref. [9], Copyright 2020 Liu et al. (**d**) Uv-Vis-NIR spectra of Cu_3_VSe_4_ nanocrystals in ethanol. Reprinted with permission from ref. [9], Copyright 2020 Liu et al.

While promising for photovoltaic applications, Cu_3_VS_4_ is not an ideal absorber material due to its indirect band gap. The efforts to improve the performance of Cu_3_VS_4_ were made by Lv et al. [4] by fabricating nanostructured Cu_3_VS_4_ as thin films (220 nm thickness). Spectroscopic ellipsometry measurements show that the refractive index of the thin films has a maximum value slightly larger than 3 at a wavelength of 720 nm, a larger value than that of either Cu_3_TaS_4_ or Cu_3_TaSe_4_ thin films made by Newhouse et al, and is close to the value of 2.7 (615 nm), calculated by using Koenigberger’s relation [58]. The extinction coefficient was also determined by spectroscopic ellipsometry, yielding a peak value between 0.6 and 0.7. Beyond 1400 nm, the thin films become transparent, as evidenced by the extinction coefficient rapidly decaying to zero after its peak value. In the visible range, the absorption coefficient was noted to be larger (>10^5^ cm^−1^) than that of the bulk form, motivating exploration into nanostructured sulvanite as an alternative to the bulk crystals as solar absorbing materials. Interestingly, Lv’s group concluded thin-film Cu_3_VS_4_ to be a near-direct band gap semiconductor, explaining that the change in band gap is likely due to size effects. A study of synthesized Cu_3_VS_4_ nanocrystals [12] showed the appearance of three peaks within the UV-vis absorption spectra. When examining the locations of the peaks between bulk and nanocrystals, Mantella et al. noted a blue shift of the peaks with decreasing the size of the nanocrystals, as shown in Figure 4a. Concurrently, a widening of the band gap with decreasing nanocrystal size was found by inspection of the density of states from DFT calculations. In Figure 4b, the absorption coefficient α is displayed versus the photon energy of Cu_3_VS_4_ nano-thin films. The change in optical behavior shown in the absorption spectra going from bulk to nanocrystals was ascribed to the effects of weak quantum confinement predicted by DFT, supporting the conclusion of Lv et al.

A recent study on the synthesis of Cu_3_VSe_4_ nanocrystals conducted by Liu et al. [9] revealed similar absorption to its sulfide partner. In a similar manner to its partner, Cu_3_VSe_4_ nanocrystals possess three absorption bands in the UV-visible range. The UV-Vis-NIR spectra of Cu_3_VSe_4_ nanocrystals, where the positions of these peaks are 391, 562, and 678 nm, corresponding to photon energies of 3.17, 2.20, and 1.83 eV, respectively, are shown in Figure 4d. The presence of these three bands indicates the IB nature of these nanocrystals, as was also found with Cu_3_VS_4_. The photoluminescence measurements (Figure 4c) show a discernible peak that depends on the excitation wavelength. The largest peak was observed at 685 nm for an excitation wavelength of 450 nm, indicating an optical band gap of 1.81 eV. This study also showed that increasing the excitation wavelength caused a redshift in the peak position, which was attributed to the distribution of both size and different emissive sites of the nanocrystals. The same group also investigated Cu_3_VSe_4_ in nanosheet form with comparable results regarding the photoluminescence and absorption bands [8].

The optical properties of the sulfide sulvanites were studied within the GGA-PBE scheme by Ali et al. [37] through the complex dielectric functional. The dielectric function curves across Cu_3_MS_4_ (M = V, Nb, Ta) exhibit three peaks that are similar but with different positions of the first two peaks, in agreement with experimental spectra. The first peak is attributed to transitions between Cu *d*-states of the valence band edge to transition metal *d*-states of the conduction band edge at the Γ-point. The second peak arises due to the transition between hybridized Cu *d*-states, S *p*-states, and transition metal *d*-states to the second conduction band. From the dielectric function, static properties were calculated; the static dielectric constant was found to be 8.56, 7.32, and 7.10 for V, Nb, and Ta, respectively, and the static refractive index was 2.93, 2.71, and 2.66. The static refractive index results for V and Ta are relatively close to the experimental values reported by Lv et al. [4] and Newhouse et al. [7].

More sophisticated methods in determining the optical properties were employed utilizing time-dependent DFT (TDDFT) within the random phase approximation (RPA) and solving the Bethe–Salpeter equation (BSE) through many-body perturbation theory by Espinosa and coworkers [3]. Their results for the imaginary part of the complex dielectric function through RPA reveal optical transitions at 3.0 eV and 3.5 eV for Cu_3_VS_4_ assigned to the Γ point, the former of which is close to that found by Ali’s group. In Cu_3_VSe_4_, the optical transitions occur at Γ and X at energies of 2.6 eV and 3.0 eV, significantly lower than those found in Cu_3_VS_4_. When the transition metal is swapped to Nb, the peak locations are found at higher energies. Cu_3_NbS_4_ has a peak at 3.5 eV from an optical transition at X and a second peak at 4.1 eV. For Cu_3_NbSe_4_, the first peak is at 2.8 eV (at R) while the second is at 4.9 eV. The results for the Ta compounds are like those of Nb; Cu_3_TaS_4_ has its first peak at 3.6 eV and Cu_3_TaSe_4_ is at 2.7 eV, both being attributed to optical transitions at Γ. The curve for the imaginary part calculated by BSE has the effect of red shifting the optical spectra of each compound and reveals continuous excitonic effects near the proximity of the first peaks. The absorption coefficients derived from BSE show large values greater than 10^5^ and as high as 10^6^. This supports measurements from Lv’s group on thin-film Cu_3_VS_4_ and a separate computational study of Cu_3_NbS_4_ [56]. Notably, these high absorption values were determined to be in the visible range for Cu_3_VS_4_, Cu_3_VSe_4_, Cu_3_NbSe_4_, and Cu_3_TaSe_4_. The remaining compounds, Cu_3_NbS_4_ and Cu_3_TaS_4_, have large absorption coefficients in the near UV range instead.

## 6. Elastic Properties

Many researchers are interested in implementing semiconductor materials into electronic devices and optical applications, such as photovoltaic cells. However, for several device applications, it is important to have information on the elastic properties of a material, such as knowing how ductile it can be for thin films. What is known about the elastic properties of the sulvanite family is largely from studies that have predicted them through DFT and the values are summarized in Table 3. The elastic properties can be derived by the calculation of the elastic constants, *C_ij_*. In the case of a cubic crystal lattice with its high symmetry, the only necessary constants are *C*_11_, *C*_22_, and *C*_44_. In terms of these elastic (stiffness) constants, the stability under elastic deformation can be expressed by the Born stability criteria [59]

$$C_{11}-C_{12}>0;C_{11}+2C_{12}>0;C_{44}>0.$$

The earliest calculation of the elastic constants was given by Osorio-Guillén et al. [60], using the procedure proposed by Erikson et al. [61] Their results showed that for the calculated elastic constants, the Born stability criteria is satisfied for each compound. Other DFT studies have found similar results for the elastic constants and have also concluded that they satisfy the stability criteria [31,37]. 

The various moduli are derived from the results of DFT by either fitting total energy versus volume data to an equation of state (Vinet, Murnaghan) or directly through the elastic constants using a method such as the Voigt–Reuss–Hill method [62]. Looking at the moduli of each compound, the sulfides are seen to have larger values than that of the selenides. Taking the values calculated using the PBEsol functional, the lowest bulk modulus *B* is found to be 39.1 GPa [60] for Cu_3_TaSe_4_ and the highest is 48.6 GPa for Cu_3_VS_4_ [57]; for the shear modulus *G*, the lowest value is 22.8 GPa and the highest is 28.66 GPa corresponding to Cu_3_VSe_4_ and Cu_3_NbS_4_, respectively; for Young’s modulus *E*, the lowest value is 57.59 GPa for Cu_3_VSe_4_ and the highest is 71.11 GPa for Cu_3_NbS_4_. The tellurides compounds have only a single study into their elastic properties and the values were determined through PBE [32]. Within the study, the tellurides compounds were shown to have moduli lower than those of the selenide compounds. It should be noted that this study has unusually high reported values in comparison to other studies, making it difficult to compare to the other values in the literature. When different DFT functionals are considered, there comes a significant change in each property. LDA, for example, predicts much larger values of the elastic constants, especially that of *C*_11_, leading to increased moduli values. On the other hand, PBE yields smaller values than either LDA or PBEsol.

In addition to the Born criteria, several studies have included Pugh’s ratio *B*/*G* [63] or Poisson’s ratio ν to characterize the response to elastic deformation. Regarding Pugh’s ratio, a critical value of approximately 1.75 separates materials into being either brittle or ductile; those with ratios below this critical value are deemed brittle while those above are ductile. Accordingly, all studies that have calculated Pugh’s ratio have found the sulvanite compounds to act as brittle materials [27,31,56,60]. Similarly, Poisson’s ratio can be used to determine if a material is brittle or ductile through a critical value of 0.33 [64] and the ratios lower than this critical value are brittle while those higher are ductile. The calculation of Poisson’s ratio concludes that these compounds are brittle, in agreement with results using Pugh’s ratio.

The overall assessment that the sulvanite compounds are brittle may prohibit the development of flexible thin films in future studies, but a definitive experimental conclusion remains to be reached.

## 7. Thermodynamic Properties

**Figure 5:**
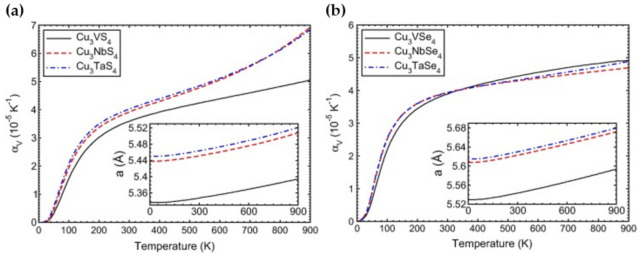
Calculated volume thermal expansion coefficient $\alpha_{V}$ versus temperature T within the quasi-harmonic approximation for the compounds Cu_3_MX_4_ (M = V, Nb, Ta; X = S, Se). (Inset) Cell parameter versus temperature. (**a**) Results for the sulfide compounds Cu_3_MS_4_. (**b**) Results for the selenide compounds Cu_3_MSe_4_. Reprinted with permission from ref. [65], Copyright 2015 Elsevier B.V.

**Figure 6:**
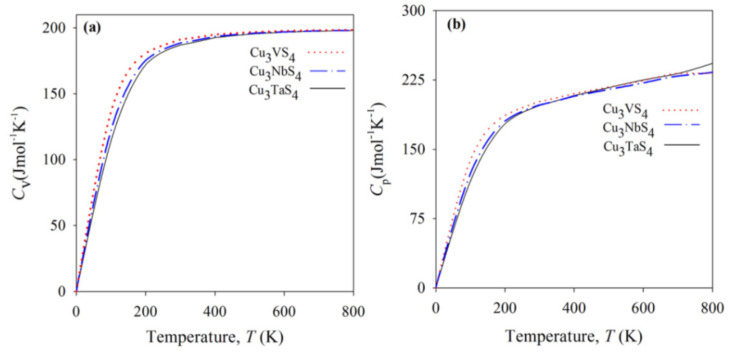
Heat capacities (**a**) at constant volume $C_{V}$ and (**b**) at constant pressure $C_{P}$ of the compounds Cu_3_MS_4_ (M = V, Nb, Ta). Reprinted with permission from ref. [27], Copyright 2014 JSR Publications, obtained 20 January 2021.

The thermodynamic properties of the sulvanite-type compounds, similar to the elastic properties, were determined via theoretical calculations. Espinosa-García et al. calculated the thermal expansion coefficient α*_V_* and heat capacity *C_V_* within the quasi-harmonic approximation (QHA) and GGA-PBEsol [65]. As shown in Figure 5, the thermal expansion coefficient for Cu_3_MX_4_ (M = V, Nb, Ta; X = S, Se) had values near 4 × 10^−5^ K^−1^ at 300 K, with Cu_3_VS_4_ being slightly under this value. Each of the compounds exhibited the rapid growth of its thermal expansion coefficient from 0 to 400 K to a value of about 4 × 10^−5^ K^−1^. A separate study done for Cu_3_NbS_4_ showed similar but larger values for the thermal expansion coefficient [56]. However, the trend of growth remained the same across both studies. The heat capacity was noted by Espinosa-García et al. to show a trend of increasing value coinciding with the transition metals (V to Nb to Ta). Each compound had its heat capacity rapidly increase from 0 K to 500 K before linearly approaching the limit of 199.5 J/mol K set by the law of Dulong and Petit at high temperatures. The calculations performed by Ali et al. also demonstrated this behavior for Cu_3_MS_4_ (M = V, Nb, Ta), concluding that the heat capacity increase with temperature is due to phonon thermal softening [27]. The results from Ali et al. are shown in Figure 6.

## 8. Recent Developments

The range of optical band gaps, *p*-type conductivity, and isotropic cubic structure makes the sulvanite-type compounds enticing for use in optoelectronic applications. Given the focus on the characterization and synthesis of these compounds, there has been less effort put forth into their potential application. This is especially true for Cu_3_MTe_4_, which has seen the least amount of published information on the sulvanite compounds, primarily due to the necessity for low-temperature solid-state methods [38] instead of the commonly used high-temperature annealing process or complications with reactivity and solubility in solution-phase methods [52]. However, the research of these compounds beyond polycrystalline powders has recently gained interest, particularly in colloidal nanocrystals [8,9,12,24]. The following section is a summary of the recent developments of these materials. 

### 8.1. Cu_3_TaS_4_, Cu_3_TaSe_4_, and Cu_3_VS_4_ Thin-Films

Cu_3_TaS_4_ and Cu_3_TaSe_4_ thin films can be fabricated onto amorphous SiO_2_ substrates using pulsed laser deposition and *ex situ* annealing, as was demonstrated by Newhouse et al. They noted that *ex situ* annealing was required, as *in situ* annealing led to phase separation or yield amorphous product [7]. Both powdered and thin-film Cu_3_TaS_4_ displayed significant photoluminescence under UV light, with a peak near 543 nm that is most intense for the powdered form. For Cu_3_TaSe_4_, the photoluminescence peaks can only be found in powders at 623 nm but not in thin films. It has been demonstrated by Hersh that it is possible to modulate the photoluminescence peak position by doping with W, resulting in a redshift of the peak [55], while still maintaining *p*-type conductivity. The intense photoluminescence and ability to modulate the peak locations offers the potential for light-emitting applications. Interestingly, Newhouse et al. proposed possible applications for these materials as transparent conducting materials, but the optical band gaps are considered too small for such uses by others [5] pointing to the fact that the final transparency would be achieved by doping [3,29].

Lv et al. prepared Cu_3_VS_4_ thin-films using pulsed laser deposition onto a glass substrate [4]. The resulting thin-films were measured to have a thickness of approximately 220 nm, which Lv and coworkers denoted as nano-thin films. This study found the absorption coefficient to be high (10^−5^ cm^−1^), with maximum values approaching 5 × 10^−5^ cm^−1^. What is most notable from the study is the reported change in electronic behavior from an indirect to near-direct band gap semiconductor. With an optical band gap of 1.35 eV and high electrical conductivity, the nano-thin film of Cu_3_VS_4_ is a possible candidate as the absorber material in thin film solar photovoltaics.

### 8.2. Cu_3_VS_4_ and Cu_3_VSe_4_ Nanocrystals

**Figure 7:**
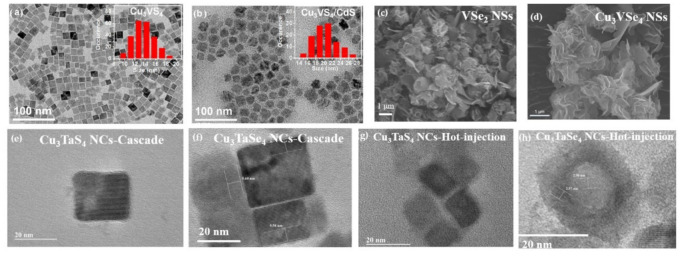
(**a**) TEM image of Cu_3_VS_4_ nanocrystals with size distribution inset. Reprinted with permission from ref. [12], Copyright 2019 American Chemical Society. (**b**) CVS/CdS nanocrystals with size distribution inset. Reprinted with permission from ref. [24], Copyright 2020 American Chemical Society. (**c**) VSe_2_ nanosheet precursor as starting material. Reprinted with permission from ref. [8], Copyright 2020 Liu et al. (**d**) Cu_3_VSe_4_ nanosheets end product. Reprinted with permission from ref. [8], Copyright 2020 Liu et al. (**e**) TEM image of Cu_3_TaS_4_ prepared by Cascade method. Reprinted with permission from ref. [10], under Creative Commons CC BY license. (**f**) TEM image of Cu_3_TaSe_4_ prepared by Cascade method. Reprinted with permission from ref. [10], under Creative Commons CC BY license. (**g**) TEM image of Cu_3_TaS_4_ prepared by hot injection. Reprinted with permission from ref. [10], under Creative Commons CC BY license. (**h**) TEM image of Cu_3_TaSe_4_ prepared by hot injection. Reprinted with permission from ref. [10], under Creative Commons CC BY license.

Colloidal Cu_3_VS_4_ nanocrystals were first reported in 2018 by Chen et al. [44] using a solution-phase method. A year later, Mantella et al. developed a similar process to produce cubic Cu_3_VS_4_ nanocrystals with tunable size [12], as shown in Figure 7a. The nanocrystals synthesized by Mantella et al. showed evidence for the existence of an intermediate bandgap, indicating the preservation of the intermediate bandgap from bulk to nanostructured. Nanocrystals of different sizes were shown to have optical spectra peaks that blueshift with decreasing particle size, something that is attributed to weak quantum confinement. However, Mantella et al. note discrepancies in predicting these peak shifts when comparing to experimental data, explaining that an in-depth study involving the surface chemistry is needed to properly describe the shifts.

More recently, two more studies involving nanocrystal sulvanite appeared. The first involves the coating of core Cu_3_VS_4_ nanocrystals with a CdS shell (CVS/CdS) by Y. Liu et al. [24], where the average CdS shell thickness is ~3 nm, as shown in Figure 7b. They showed that normal Cu_3_VS_4_ nanocrystals have a plasmonic-like, quasi-static resonance in the UV-visible spectrum that is brought on by IB within the electronic structure. Upon coating, the CVS/CdS nanocrystal was shown to change from plasmonic-like to excitonic. Liu et al. proposed that the mechanism of transition from plasmonic-like to excitonic nanocrystals is a result of the mixing of IB states from the Cu_3_VS_4_ core and the conduction band of the CdS shell. 

The second study is the first report of the synthesis of colloidal Cu_3_VSe_4_ nanocrystals [9]. In this study, the Cu_3_VSe_4_ nanocrystals were synthesized by a solution-phase process similar to that described by Chen et al. The synthesized nanocrystals exhibited excitation-dependent photoluminescence, the existence of intermediate bandgap and possessed an optical band gap of 1.81 eV. Additionally, the photoelectrochemical measurements of Cu_3_VSe_4_ nanocrystal-dispersed thin films revealed a stable *p*-type photocurrent (4 μA/cm^2^) in KCl solution. Both Cu_3_VS_4_ and Cu_3_VSe_4_ nanocrystals, with their IB and optical band gaps, show potential applications for photovoltaic applications.

### 8.3. Cu_3_VSe_4_ Nanosheets

According to literature, 2D nanomaterials have specific properties compared to 0D, 1D and 3D nanomaterials, which impact their performance in various applications. For instance, the large lateral dimensions and atomic thickness of 2D materials provide an ultrahigh specific surface area which is ideal in surface-related catalysis. For example, a single Fe site confined in a graphene matrix could efficiently catalyze benzene oxidation at room temperature [66]. Besides, the confinement of electron-hole pair in 2D semiconductors influences their electric and optoelectronic properties and improves their photovoltaic performance in thin-film solar cells [67]. Furthermore, in photocatalysis, it was proved that the preparation of plasmonic photocatalysts with improved light harvesting, reduced carrier recombination, and thus, improved photocatalytic activity benefit from nano-architectures. Among such morphologies, faceted nanoparticles, nanotubes, aerogels, and other nanostructures of semiconductors showed improved photocatalytic activity and stability [68].

Following the trend of investigating sulvanite in nanostructured form, new synthesis methods for producing Cu_3_VSe_4_ nanosheets were recently reported by Liu and coworkers [8]. Using a templating method, binary VSe_2_ nanosheets are inserted with Cu cations to produce the ternary compound Cu_3_VSe_4_ nanosheets, as shown in Figure 7c,d. Similar to the Cu_3_VSe_4_ nanocrystals, these nanosheets exhibit three absorption peaks that have become characteristic of nanostructured sulvanite. These peaks are attributed to the IB nature of the electronic structure. The PL measurements showed intense emission peaks at 690 nm, yielding an optical band gap of 1.80 eV; this is similar to the nanocrystals investigated earlier by the same group. The photocurrent measurements of thin-films made from Cu_3_VSe_4_ nanosheets demonstrated *p*-type conductivity. Notably, the Cu_3_VSe_4_ nanosheets-FTO thin-films exhibited a nine-fold improvement of the photocurrent produced (~0.036 mA cm^−2^) when compared to an earlier study on Cu_3_VSe_4_ nanocrystals-FTO thin-films, a fact linked to the higher surface area that is common with 2D morphologies. An electrochemical cell was fabricated from Cu_3_VSe_4_ nanocrystals-FTO thin-films, exhibiting a charge transfer resistance of 300 Ω cm^2^.

The Cu_3_VSe_4_ nanosheets coated in thin films have potential for photovoltaic applications. Beyond this, the 2D morphology allows for other applications, such as wearable electronics, biosensors and internet-of-things (IoT) applications.

### 8.4. Cu_3_TaS_4_, and Cu_3_TaSe_4_ Nanocrystals

The first solution-phase syntheses of Cu_3_TaS_4_ and Cu_3_TaSe_4_ nanocrystals were reported by Liu et al. in [10]. In this work, the nanosized Cu_3_TaS_4_ nanocrystals with prismatic morphology were prepared via two solution-phase methods, involving a novel cascade approach and traditional hot-injection method. The cascade method is composed of the preparation of TaS_2_ nanoflakes and the incorporation of Cu cations into the starting template of TaS_2_, while the hot-injection method is the injection of sulfur source into the hot Cu-Ta cation precursors. As shown in Figure 7e,g, the Cu_3_TaS_4_ products prepared using the cascade approach and hot-injection method formed a cubic shape with an average size of around 20 and ~15 nm, respectively. In the same work, the Cu_3_TaSe_4_ nanocrystals were obtained via the same cascade method, where the Cu cations were introduced into the formed TaSe_2_ nanoflakes, as shown in Figure 7f. However, when using the same hot-injection method to prepare Cu_3_TaSe_4_ materials, the resulting product is Cu_3_TaSe_4_ core-shells, as shown in Figure 7h. The synthesized Cu_3_TaS_4_ and Cu_3_TaSe_4_ nanocrystals showed good absorption in the ultraviolet-visible region; however, the Cu_3_TaSe_4_ core-shells exhibited broad absorption bands in the UV-Vis and the near-infrared region. Moreover, they investigated the PL of Cu_3_TaS_4_ and Cu_3_TaSe_4_ nanocrystals, which revealed their optical bandgaps of 2.54 and 2.32 eV, respectively. A Au/Cu_3_TaS_4_-glass/Au device was fabricated to proves the electrical conductivity of Cu_3_TaS_4_ nanocrystals.

### 8.5. Photocatalysts and Photoelectrodes from Cu_3_MS_4_

The sulvanite-type compounds were investigated by Takayama et al. for their ability to serve as photocatalysts for sacrificial H_2_ evolution. In their report, Cu_3_MS_4_ (M = V, Nb, Ta) all displayed continuous H_2_ production under visible light irradiation [41]. Cu_3_NbS_4_ and Cu_3_TaS_4_ both showed H_2_ evolution without the need for a cocatalyst while Cu_3_VS_4_ is unable to show activity without a cocatalyst. With the addition of a Ru-cocatalyst, the H_2_ evolution approximately doubled for Cu_3_NbS_4_ and Cu_3_TaS_4_. Of the three, Cu_3_NbS_4_ was reported to have the highest rate of H_2_ evolution, but Cu_3_VS_4_, despite its lower values, was able to show activity even within the near-IR region (800 nm).

A later contribution by the same group expanded their study to include Cu_3_Nb_1−x_V_x_S_4_ and Cu_3_Ta_1−x_V_x_S_4_ [11]. Cu_3_MS_4_ once again successfully showed photocatalytic capabilities along with Cu_3_Nb_1−x_V_x_S_4_ and Cu_3_Ta_1−x_V_x_S_4_ plus Ru-cocatalyst. Interestingly, the mixing of transition metals for Cu_3_Nb_1−x_V_x_S_4_ and Cu_3_Nb_1−x_V_x_S_4_ resulted in much higher H_2_ evolution than just pure sulvanite compound. Cu_3_NbS_4_ provided a rate of H_2_ evolution of 537 μmol/h and Cu_3_Nb_0.9_V_0.1_S_4_ had almost double the value at 1090 μmol/h. Similarly, Cu_3_TaS_4_ had just 19 μmol/h, while Cu_3_Ta_0.7_V_0.3_S_4_ was much larger at 802 μmol/h. These compounds were also used as photoelectrodes within the same study. When illuminated by visible light, Cu_3_Nb_1−x_V_x_S_4_ and Ru-loaded Cu_3_Nb_1−x_V_x_S_4_ both exhibited cathodic photocurrents.

These findings indicate that the sulvanite-type compounds can potentially be used in water-splitting applications.

## 9. Conclusions

Much progress into the study and applications of the sulvanite-type compounds was made since their initial characterization by Pauling and Hultgren. Despite the number of publications that are available, much of our current understanding of these unique materials are derived from fundamental measurements and DFT predictions. Thus, there is a need for more experimental studies to fully unleash the theoretically predicted potential into the outlined applications. This is most apparent for the lesser investigated telluride compounds, of which almost all information for them comes from theoretical calculations. The optical band gaps and large absorption of many sulvanites suggest their promise for photovoltaic applications. The thin films made from the sulvanite-type compounds at the nanoscale demonstrate great potential for photovoltaic applications, as shown by the most recent studies related to their nanocrystal and nanosheet morphologies. Newer studies have revealed the possibility for use of sulvanites in water-splitting, presenting the chance for additional alternative energy applications. With the recent development of synthesis techniques for sulvanite nanocrystals and nanosheets, IB materials could be further explored in solar photovoltaics. With the recent interest from the scientific community, it is anticipated that better insight into these materials will be gained in the near future.

## Figures and Tables

**Table 1:** Experimental and theoretical cell parameters *a*. Notes: * PBEsol + U, ^†^ WC.

	*a* [Å]		*a* [Å]			
	Experimental		PBE	PBEsol	HSE06	Other
Cu_3_VS_4_	5.36 [24]	5.391 [25]	5.41 [23]	5.31 [26]	5.46 [6]	
	5.37 [13]	5.3912 [16]	5.4213 [27]	5.358 [5] *		
	5.384 [28]	5.3918 [4]	5.4374 [29]			
	5.39 [15]	5.393 [30]				
	5.391 [14]	5.4675 [4]				
Cu_3_VSe_4_	5.569 [14]		5.62 [23]	5.51 [26]	5.672 [6]	5.53 [31] ^†^
	5.559 [28]		5.65 [32]	5.557 [5] *		
	5.5636 [18]					
	5.57 [15]					
Cu_3_VTe_4_	5.859 [14]		5.93 [23]	5.838 [5] *	5.988 [6]	
			5.95 [32]			
Cu_3_NbS_4_	5.494 [28]		5.5292 [27]	5.41 [26]	5.572 [6]	
	5.495 [33]		5.5492 [29]	5.472 [5] *		
	5.5 [14,15,25]					
	5.5001 [19]					
	5.501 [34]					
Cu_3_NbSe_4_	5.654 [14]		5.73 [32]	5.59 [26]	5.746 [6]	5.6372 [31] ^†^
	5.638 [17]			5.641 [5] *		
	5.65 [15]					
	5.655 [34]					
	5.657 [28]					
Cu_3_NbTe_4_	5.923 [14]	5.525 [25]	6.00 [32]	5.902 [5] *	6.03 [6]	
	5.9217 [21]					
Cu_3_TaS_4_	5.514 [14]		5.5588 [29]	5.43 [26]	5.584 [6]	
	5.506 [28]		5.5622 [27]	5.480 [5] *		
	5.5145 [35]					
	5.5185 [36]					
	5.52 [15]					
Cu_3_TaSe_4_	5.664 [14]	5.688 [28]	5.74 [32]	5.59 [26]	5.753 [6]	5.641 [31] ^†^
	5.66 [20]			5.650 [5] *		
	5.6613 [37]					
	5.6625 [36]					
	5.67 [15]					
Cu_3_TaTe_4_	5.928 [14]		6.01 [32]	5.906 [5] *	6.033 [6]	
	5.9283 [36]					
	5.93 [38]					

**Table 2:** Experimental and calculated band gaps for the sulvanite family of compounds. Notes: * TB-mBJ, † PBEsol + G_0_W_0_, α GGA-WC, β GGA-EV.

Compound	$E_{g}(\mathrm{eV})$	$E_{g}\;(\mathrm{eV})\;\mathrm{Fundamental}$					
	Experimental	PBE	PBEsol	PBEsol + U	HSE06	Other	
Cu_3_VS_4_	1.3 [25]	1.02 [29,54]	1.04 [3]	1.13 [5]	2.05 [6]	1.04 [11]	
	1.35 [4]	1.03 [23]			2.07 [3]	2.26 [3] ^†^	
	1.55 [11]	1.041 [27]					
Cu_3_VSe_4_	1.81 [9]	0.82 [23]	0.81 [3]	0.87 [5]	1.73 [3]	0.820 [31] ^α^	1.086 [31] *
	1.80 [8]	0.829 [32]			1.76 [6]	0.96 [31] ^β^	1.91 [3] ^†^
						1.061 [32] *	
Cu_3_VTe_4_	-	0.57 [23]		0.53 [5]	1.23 [6]	0.769 [32] *	
		0.592 [32]					
Cu_3_NbS_4_	2.50 [11]	1.64 [54]	1.64 [3]	1.82 [5]	2.66 [3,6]	1.69 [11]	
	2.56 [55]	1.66 [29]	1.65 [56]			3.01 [3] ^†^	
	2.6 [34]	1.667 [27]					
Cu_3_NbSe_4_	2.13 [55]	1.376 [32]	1.32 [3]	1.45 [5]	2.20 [3]	1.36 [31] ^α^	1.53 [31] *
	2.14 [53]				2.24 [6]	1.52 [31] ^β^	2.24 [3] ^†^
	2.2 [34]					1.520 [32] *	
Cu_3_NbTe_4_	-	0.976 [32]		0.92 [5]	1.62 [6]	1.086 [32] *	
Cu_3_TaS_4_	2.54 [10]	1.815 [27]	1.88 [3]	2.10 [5]	2.94 [3]	1.84 [11]	
	2.70 [7]	1.94 [29]			2.97 [6]	3.19 [3] ^†^	
	2.75 [55]	1.91 [54]					
	2.83 [11]						
Cu_3_TaSe_4_	2.32 [10]	1.611 [32]	1.54 [3]	1.71 [5]	2.47 [3]	1.581 [31] ^α^	1.845 [31] *
	2.35 [7]				2.52 [6]	1.798 [31] ^β^	2.38 [3] ^†^
	2.36 [55]					1.828 [32] *	
	2.43 [53]						
Cu_3_TaTe_4_	-	1.171 [32]		1.11 [5]	1.84 [6]	1.323 [32] *	

**Table 3:** Elastic properties of the sulvanite compounds as determined from DFT including the elastic constants $C_{11},C_{12},C_{44}$, bulk modulus B, shear modulus G, Young’s modulus E, the Pugh ratio B/G, and Poisson’s ratio ν. Units are in GPa. Notes: * LDA, ^†^ WC.

Compound		C_11_	*C*_12_	*C*_44_	B	G	E	B/G	ν
Cu_3_VS_4_	PBE	92.1 [60]	17.3 [60]	20.4 [60]	42.2 [60]	27.08 [60]	66.92 [60]	1.56 [60]	0.20 [27]
		92.4 [27]	16.0 [27]	26.2 [27]	41.4 [27]	30.5 [27]	73.40 [27]	1.36 [27]	
	PBEsol	104.8 [60]	20.4 [60]	21.0 [60]	48.6 [26,60]	27.85 [60]	70.14 [60]	1.74 [60]	
	Other	115.7 [60] *	23.7 [60] *	22.3 [60] *	54.4 [60] *	29.93 [60] *	75.88 [60] *	1.82 [60] *	
Cu_3_NbS_4_	PBE	90.81 [57]	11.98 [57]	17.95 [57]					
		91.6 [60]	12.0 [60]	17.9 [60]	38.5 [60]	24.54 [60]	60.72 [60]	1.57 [60]	
		97.8 [27]	15.6 [27]	22.3 [27]	43.0 [27]	28.5 [27]	70.00 [27]	1.51 [27]	0.22 [27]
	PBEsol	107.0 [60]	15.1 [60]	20.7 [60]	45.7 [60]	28.66 [60]	71.11 [60]	1.59 [60]	
		105.77 [56]	14.33 [56]	20.05 [56]	44.81 [56]	28.08 [56]	69.68 [56]	1.59 [56]	0.24 [56]
					45.2 [26]				
	Other	118.0 [60] *	18.2 [60] *	22.1 [60] *	51.5 [60] *	30.86 [60] *	77.18 [60] *	1.66 [60] *	
Cu_3_TaS_4_	PBE	88.40 [57]	12.49 [57]	17.83 [57]					
		89.0 [60]	12.6 [60]	17.5 [60]	38.1 [60]	24.06 [60]	59.62 [60]	1.58 [60]	
		96.2 [27]	11.0 [27]	23.6 [27]	39.4 [27]	30.0 [27]	71.70 [27]	1.31 [27]	0.19 [27]
	PBEsol	104.6 [60]	15.2 [60]	20.4 [60]	45.0 [60]	28.12 [60]	69.82 [60]	1.60 [60]	
					44.4 [26]				
	Other	115.8 [60] *	18.4 [60] *	21.9 [60] *	50.9 [60] *	30.39 [60] *	76.03 [60] *	1.67 [60] *	
Cu_3_VSe_4_	PBE	69.8 [60]	16.3 [60]	19.1 [60]	34.3 [60]	21.10 [60]	52.54 [60]	1.63 [60]	0.24 [32]
		109.52 [32]	52.83 [32]	57.80 [32]	71.73 [32]	43.42 [32]			
	PBEsol	81.1 [60]	20.2 [60]	18.8 [60]	40.5 [60]	22.80 [60]	57.59 [60]	1.77 [60]	
					40.6 [26]				
	Other	83.46 [31] ^†^	25.69 [31] ^†^	29.98 [31] ^†^	44.94 [31] ^†^	29.54 [31] ^†^	72.68 [31] ^†^	1.52 [31] ^†^	
		92.1 [60] *	24.2 [60] *	21.7 [60] *	46.8 [60] *	26.02 [60] *	65.88 [60] *	1.80 [60] *	0.23 [60] ^†^
Cu_3_NbSe_4_	PBE	73.20 [57]	12.90 [57]	17.50 [57]					
		74.6 [60]	13.5 [60]	18.3 [60]	33.9 [60]	22.50 [60]	55.26 [60]	1.50 [60]	
		116.31 [32]	52.25 [32]	53.54 [32]	73.60 [32]	43.52 [32]			0.25 [32]
	PBEsol	85.9 [60]	16.4 [60]	19.9 [60]	39.6 [60]	24.91 [60]	61.76 [60]	1.59 [60]	
					40.2 [26]				
	Other	95.7 [60] *89.28 [31] ^†^	19.5 [60] *21.89 [31] ^†^	19.9 [60] *32.05 [31] ^†^	44.9 [60] *43.44 [31] ^†^	25.88 [60] *32.70 [31] ^†^	65.14 [60] *78.38 [31] ^†^	1.73 [60] *1.33 [31] ^†^	0.20 [31] ^†^
Cu_3_TaSe_4_	PBE	70.40 [57]	13.20 [57]	17.40 [57]					
		72.4 [60]	14.1 [60]	18.2 [60]	33.6 [60]	21.99 [60]	54.14 [60]	1.53 [60]	
		116.44 [32]	55.89 [32]	54.87 [32]	76.08 [32]	43.22 [32]			0.26 [32]
	PBEsol	83.5 [60]	16.9 [60]	20.0 [60]	39.1 [60]39.3 [26]	24.57 [60]	60.94 [60]	1.59 [60]	-
	Other	90.70 [31] ^†^	19.2 [31] ^†^	33.54 [31] ^†^	43.03 [31] ^†^	34.41 [31] ^†^	81.5 [31] ^†^	1.25 [31] ^†^	0.18 [31] ^†^
		93.2 [60] *	19.7 [60] *	22.2 [60] *	44.2 [60] *	27.23 [60] *	67.76 [60] *	1.62 [60] *	
Cu_3_VTe_4_	PBE	86.59 [32]	47.35 [32]	53.42 [32]	60.43 [32]	35.76 [32]			0.25 [32]
Cu_3_NbTe_4_	PBE	94.26 [32]	47.84 [32]	52.50 [32]	63.31 [32]	37.83 [32]			0.25 [32]
Cu_3_TaTe_4_	PBE	93.81 [32]	50.82 [32]	53.69 [32]	65.15 [32]	37.19 [32]			0.26 [32]
